# Comparative disinfection efficacy in aquaculture: novel methylene bis(thiocyanate) vs. conventional trichloroisocyanuric acid against *Aeromonas hydrophila*

**DOI:** 10.3389/fmicb.2025.1611576

**Published:** 2025-07-21

**Authors:** Guang Yang, Ying Huang, Ning Ma, Kai Li, Xiao-mei Wang, Lian-bo Zhang, Wen-bo Yang, Wan-li Zhang, Lei Xia, Hong-Yu Zhang, Li-lai Yuan

**Affiliations:** ^1^Fisheries Engineering Institute, Chinese Academy of Fishery Sciences, Beijing, China; ^2^Fishery Resource and Environment Research Center, Chinese Academy of Fishery Sciences, Beijing, China; ^3^Chinese Academy of Fishery Sciences, Beijing, China

**Keywords:** *Aeromonas*, methylene bis (thiocyanate), trichloroisocyanuric acid, disinfection, aquaculture

## Abstract

*Aeromonas hydrophila* is a major pathogen responsible for significant economic losses in global aquaculture. Inhibitory and bactericidal activities of seven disinfectants were tested against 10 aquatic pathogens, including *A. hydrophila*, *A. veronii*, *A. salmonicida*, *A. sobria*, *Edwardsiella tarda*, *E. ictaluri*, *Pseudomonas aeruginosa*, and *Yersinia ruckeri*. Minimal effective concentrations were determined via quantitative suspension tests, while a scale-trauma zebrafish model assessed *in vivo* protection. Methylene bis(thiocyanate) (MBT) exhibited the strongest antimicrobial activity, with minimum inhibitory concentrations and minimum bactericidal concentrations of 0.1–0.4 mg/L and 0.2–0.8 mg/L, respectively. At 0.01 mg/L, MBT achieved a 3-log pathogen inactivation, equivalent to 0.2 mg/L Trichloroisocyanuric acid (TCCA). *In vivo*, 0.01 mg/L MBT provided 100% protection in zebrafish after 6 and 12 h exposure, whereas 0.2 mg/L TCCA yielded 95.83 ± 3.61% survival after 1 h. The safe concentrations of MBT and TCCA for zebrafish were 0.0364 and 0.0677 mg/L, respectively. The results showed that both TCCA and MBT effectively controlled *A. hydrophila* infection; however, MBT demonstrated greater potential for aquaculture applications due to its lower effective concentration (0.01 mg/L) and reduced sensitivity to interference from organic matter. In addition, this study presents a systematic protocol for evaluating the efficacy of disinfectants.

## Introduction

Inland aquaculture has reached a high level of intensification, however, the frequent occurrence of aquatic diseases remains a significant challenge in the cultivation process. Among various pathogens, *Aeromonas hydrophila* is one of the most prevalent and dominant, posing a major threat to global aquaculture and causing substantial economic losses worldwide (Dien et al., 2022; Borella et al., 2020; El-Gohary et al., 2020; Ran et al., 2018).

Disinfectants and antibiotics remain the primary means for preventing and controlling bacterial diseases in aquaculture. However, the increasing threat of antimicrobial resistance to both ecosystems and human health has led to stricter regulations on the types and quantities of antibiotics permitted in aquaculture, further heightening the demand for effective disinfectants.

Trichloroisocyanuric acid (TCCA) is one of the widely used traditional disinfectants in aquaculture production. Despite its prevalence, research on controlling aquatic pathogens remains limited, with existing studies predominantly focusing on minimum inhibitory concentration (MIC), minimum bactericidal concentration (MBC), and acute toxicity to fish. Field-based disinfection assessments typically prioritize reductions in total heterotrophic bacterial counts (Zhang et al., 2023), while systematic evaluations of pathogen-specific efficacy—particularly against *A. hydrophila*—remain critically underexplored.

Methylene bis(thiocyanate) (MBT), a broad-spectrum biocide, has been extensively utilized in industrial applications, including latex emulsion preservation, paint production, wood treatment, leather processing, and cooling water systems, where it demonstrates potent inhibitory effects against algae, fungi, and sulfate-reducing anaerobic *Desulfovibrio* species (Maas-Diepeveen and van Leeuwen, 1988; Singh, 2008). In China, MBT emulsifiable concentrate was temporarily employed as an aquaculture disinfectant but was discontinued in 2006 due to insufficient scientific validation of its efficacy and safety. Recent studies have shown that *Saprolegnia parasitica,* a destructive aquatic pathogen, is highly susceptible to MBT (Wang et al., 2019). Additionally, Chen et al. (2021) reported its strong antibacterial activity against three strains of *A. hydrophila*. However, these studies have focused solely on MIC and MBC values, which is insufficient to fully establish MBT as a reliable disinfectant for controlling *A. hydrophila* in aquaculture settings.

This study systematically evaluates the antibacterial and protective efficacy of TCCA and MBT against *A. hydrophila* through integrated *in vitro* and *in vivo* investigations. Additionally, the toxicity of MBT to aquatic species is assessed to determine its feasibility for aquaculture applications. The findings of this research will provide valuable insights into the prevention and control of bacterial diseases caused by *A. hydrophila* and contribute to the development of effective, sustainable disinfection strategies in aquaculture.

## Materials and methods

### Bacterial strains and growth conditions

Ten bacterial strains used for disinfectant testing were either isolated from diseased freshwater fish or generously provided by Drs. Yibin Yang and Yong Zhou of the Yangtze River Fisheries Research Institute of the Chinese Academy of Fishery Sciences (Table 1). The strains were cultured overnight at 28°C in nutrient broth (AOBOX, Beijing) with continuous shaking at 180 rpm. Cell density was measured spectrophotometrically at 600 nm, and the bacterial concentration was adjusted to 2 × 10^3^–10^6^ cfu/mL for subsequent experiments.

**Table 1:** Sources of the bacterial isolates used in this study.

Number	Species	Code	Source
1	*Aeromonas hydrophila*	Ah0548	*Carassius auratus*
2	*Aeromonas hydrophila*	Ah296	*Megalobrama amblycephala*
3	*Aeromonas veronii*	AvB1	*Cyprinus carpio koi*
4	*Aeromonas veronii*	Av0503	*Ctenopharyngodon idella*
5	*Aeromonas salmonicida*	AsH1	*Cyprinus carpio koi*
6	*Aeromonas sobria*	AsoH2	*Ctenopharyngodon idella*
7	*Edwardsiella tarda*	EtZ1	*Pelteobagrus fulvidraco*
8	*Edwardsiella ictaluri*	Ei9	*Ictalurus punctatus*
9	*Pseudomonas aeruginosa*	Pa22	*Ictalurus punctatus*
10	*Yersinia ruckeri*	Yr36	*Ictalurus punctatus*

### Experimental disinfectants

The disinfectants used in this study are listed in Table 2. Stock solutions were prepared according to Clinical and Laboratory Standards Institute guidelines (CLSI, 2018). MBT was dissolved in dimethyl sulfoxide to achieve a 1% (w/v) stock solution. The remaining six disinfectants—PVP-I (1% w/v), H_2_O_2_ (0.1% w/v), PAA (0.01% w/v), BB (1% w/v), TCCA (0.1% w/v), and GA (1% w/v)—were prepared as stock solutions using deionized water. These stocks were then diluted with deionized water to 10 × the final effective concentrations specified in Table 2 for MIC and MBC testing.

**Table 2:** Disinfectants information for the efficacy evaluation.

Disinfectants	Abbreviation	Source	Concentrations of effective constituent used (mg/L)
Methylene dithiocyanate	MBT	Aladdin Chemistry Co., Ltd	0, 0.05, 0.1, 0.2, 0.4, 0.8, 1.6, 3.2
Povidone-iodine	PVP-I	Shanghai Macklin Biochemical Technology Co., Ltd	0, 10, 50, 100, 200, 300, 600, 1,000
Hydrogen peroxide	H_2_O_2_	Shanghai Macklin Biochemical Technology Co., Ltd	0, 8, 16, 24, 32, 40, 48, 56, 64, 72, 80
Peracetic acid	PAA	Shandong Annjet High-Tech Disinfection Technology Co., Ltd.	0, 0.1, 0.5, 1.0, 1.5, 2.0, 2.5, 5, 10
Benzalkonium bromide	BB	Shanghai Macklin Biochemical Technology Co., Ltd	0, 5, 10, 20, 40, 80, 120
Trichloroisocyanuric acid	TCCA	Hebei Balingwei Hyperfine Material Co., Ltd	0, 10, 20, 40, 60, 80, 100, 120, 140
Glutaraldehyde	GA	Shanghai Macklin Biochemical Technology Co., Ltd	0, 20, 40, 60, 80, 100, 120, 140, 160

### Experimental animals

The zebrafish were obtained from Beijing High Education Research Technology Co., Ltd., with standardized body lengths of 2.12 ± 0.09 cm (mean ± SD). The crucian carp were obtained from Beijing Longchi Aquaculture Farm, with body lengths of 2.33 ± 0.18 cm (mean ± SD). All species underwent 14-day acclimatization period in aquatic tanks prior to the experiments. The water conditions throughout experiments were maintained at 25 ± 3°C, pH 7.1–7.8, with constant aeration (>6.5 mg/L dissolved oxygen). During acclimatization, the fish were fed twice daily with commercial fish feed.

### Antimicrobial tests

#### Inhibitory and bactericidal activities of seven disinfectants

The susceptibilities of the 10 bacterial strains to the seven disinfectants were determined using a microdilution method in 96-well microtiter plates, following the Clinical and Laboratory Standards Institute (CLSI) M07 (CLSI, 2018) and Zou et al. (2020), with some modifications. The disinfectant stock solutions were diluted with distilled water, and 10 μL of each diluted solution was dispensed into the wells, yielding the final concentrations listed in Table 2. Next, 90 μL of the prepared bacterial culture was added to the wells to achieve a final concentration of 5 × 10^5^ to 1 × 10^6^ cfu/mL, and the plates were immediately incubated at 28 ± 1°C under constant agitation (300 rpm). To minimize evaporation, peripheral wells were excluded from the assay and filled with 100 μL of sterile water to buffer against edge effects. Only the inner wells were used for samples, and a non-fan-assisted incubator was employed. The minimum inhibitory concentration (MIC) was determined after 24 h. Cultures without antimicrobial agents served as positive controls, whereas nutrient broth without bacteria was used as a negative control.

The minimum bactericidal concentration (MBC) was determined using the tube dilution method (Zou et al., 2020). The four lowest concentrations that showed no significant growth were diluted 1:20 in fresh nutrient broth and subcultured to assess bacterial survival. The lowest concentration at which no bacterial growth was observed was recorded as the MBC. All experiments were performed in triplicate to confirm reproducibility, in accordance with CLSI guidelines.

#### Comparison of bactericidal activity between MBT and TCCA in suspension tests

A quantitative suspension test was performed following BS EN 13727:2012 + A2:2015 (British Standards Institution, 2015) and Verner-Jeffreys et al. (2009), with some modifications. MBT and TCCA solutions were prepared in hard water (1.248 mM/L MgCl_2_, 3.328 mM/L CaCl_2_, 2.496 mM/L NaHCO_3_; pH 7.0 ± 0.2) at a 10 × concentration prior to use. The final concentrations tested were 0.005, 0.01, 0.015, 0.02, and 0.04 mg/L for MBT, and 0.05, 0.1, 0.2, 0.4 and 0.6 for TCCA. Strain Ah0548 was used for the *in vitro* assays. The bacteria were inoculated into fresh nutrient broth at a ratio of 1:1000 and cultured overnight at 28°C under constant agitation (180 rpm) for 12–18 h. Subsequently, 1 mL of bacterial culture (1–4 × 10^9^ cfu/mL, determined by plate count) was centrifuged at 5000 × *g* for 5 min. The pellet was washed once with 1 mL of hard water and then resuspended in 1 mL of hard water. Finally, the suspension was diluted with hard water to achieve a 10 × working concentration corresponding to approximate 2 × 10^6^ cfu/mL. A total 100 μL of this bacterial suspension was added to 900 μL of the diluted disinfectant solution (prepared by diluting 100 μL of the 10 × disinfectant in hard water). In the control groups, 100 μL of hard water was added in place of the disinfectant. After the required contact times (1, 6, 12, and 24 h for MBT, and 30 min, 1 h, and 6 h for TCCA), 100 μL samples were serially diluted and plated to nutrient agar. The plates were then incubated at 28°C for 24–96 h, and the resulting colonies were counted.

### Protective effect of MBT and TCCA against *A. hydrophila*

#### Establishment of scale-trauma immersion infection model

Strain Ah0548 was used for the challenge test, which was conducted in 10 L tanks. Prior to the immersion infection experiment, the experimental fish were fasted for 48 h and anesthetized with 40 mg/L eugenol (MACKLIN, Shanghai). One side of each fish was then scraped, and the fish were promptly returned to the experimental tanks. After recovery, the fish were transferred to tanks designated for the infection and control groups.

For the immersion infection test, four concentrations of *A. hydrophila* (2 × 10^3^, 2 × 10^4^, 2 × 10^5^, and 2 × 10^6^ cfu/mL) were used to form the infection group. Tanks without pathogenic bacteria were designated as the scale-trauma control group, while tanks with neither pathogenic bacteria nor scale-trauma operations were served as the common control group. Each group consisted of three replicates, with each replicate containing 16 zebrafish.

Additionally, a crucian carp immersion infection test was performed using the same method at a concentration of 2 × 10^4^ cfu/mL.

#### Protective effects of MBT and TCCA on zebrafish and crucian carp

In this experiment, two treatment groups, an infection control group, and a common control group were established. In the treatment groups, a bacterial suspension of Ah0548 was added to the tanks to achieve a final density of 2 × 10^4^ cfu/mL, along with MBT or TCCA to reach final effective concentrations of 0.01 and 0.2 mg/L, respectively. The infection control group received only the bacterial suspension (2 × 10^4^ cfu/mL), while the common control group consisted of fish with scale trauma but without exposure to Ah0548 or disinfectants.

Zebrafish and crucian carp with one-side scale trauma were transferred at 1, 6, and 12 h post-disinfection, and fish mortality was recorded at 12, 24, 48, and 96 h. Additionally, liver, intestine, and muscle samples were collected from the fish for histological examination.

Furthermore, the protective effects of MBT (0.01 mg/L) and TCCA (0.2 mg/L) were also evaluated in crucian carp after 6 h of exposure using the same protocol described above.

After euthanizing the experimental zebrafish, the viscera from both the control and treatment groups were aseptically removed. The dissected organs were placed in 2.0-mL tubes containing 300 μL of phosphate-buffered saline (PBS, pH 7.2) and homogenized using a homogenizer (WiseTis®HG-15A, DAIHAN). The homogenates were subsequently analyzed by PCR for the *aerA* virulence gene of *A. hydrophila*, using primer *aerA* (A1:5′-CAAGAACAAGTTCAAGTGGCCA-3′; and A2:5′ACGAAGGTGTGGTTCCAGT-3′) as described by Wang et al. (2003).

#### Acute toxicity tests

A static non-renewable acute toxicity bioassay was conducted following OECD Test Guideline 2023 (OECD, 2019; Kumar et al., 2011) to determine the median lethal concentration (LC_50_) of MBT and TCCA for zebrafish and crucian carp after 96 h of exposure. Fish were exposed to a series of MBT concentrations (0.1, 0.14, 0.2, 0.28, 0.4, 0.56 and 0.8 mg/L) and TCCA concentrations (0.2, 0.27, 0.36, 0.49, 0.66, 0.89, 1.2 mg/L) in a geometric progression, based on the preliminary experimental results.

The MBT control group was exposed to dimethyl sulfoxide at a solvent volume equivalent to the maximum concentration employed in test solution preparation, while the TCCA control group received no treatment. All experimental groups were replicated three times (15 fish per tank), and fish were fasted for the duration of the trial. The number of dead fish was recorded at 24, 48, 72, and 96 h post-dosing, with dead individuals promptly removed from each tank. Safe concentrations (SC) were calculated by multiplying 96-h LC_50_ by an empirical factor of 0.1 (Sprague, 1971; Zou et al., 2020). Data from the experiments were analyzed using probit analysis in SPSS 26.0.

#### Calculations and statistical analysis

Relative reduction (R) and log reduction were calculated according to the modified BS EN 13727:2012 + A2:2015 (British Standards Institution, 2015; Verner-Jeffreys et al., 2009). For each disinfectant concentration and experimental condition, the decimal log reduction (lgR) was calculated using the formula:


$$\mathrm{lgR}={\mathrm{lgN}}_{0}–{\mathrm{lgN}}_{a}$$

Where R is the reduction in viability, N_0_ is the number of cells per mL in the test mixture at the start of the contact time (time “zero” = 0), and N_a_ is the number of survivors per mL at the end of contact time.

Cumulative mortality and survival rate data were expressed as means±SD. Data were analyzed using one-way analysis of variance (ANOVA) in SPSS 26.0, and the least significant difference (LSD) test was applied to detect differences between groups. Statistical significance was set at a *p* < 0.05.

## Results

### Comparative antimicrobial efficacy of disinfectants against pathogenic bacteria

The MICs and MBCs of seven disinfectants against 10 pathogenic bacterial strains are presented in Tables 3, 4. MBT demonstrated superior antimicrobial performance, exhibiting both inhibitory and bactericidal effects across all tested strains at concentrations ranging from 0.1 to 0.8 mg/L. PAA showed the second highest efficacy, with MIC and MBC values between 1 and 5 mg/L. Three disinfectants - H_2_O_2_, BB, and TCCA - displayed comparable antimicrobial profiles, maintaining MIC/MBC values of 20–80 mg/L for most strains, except that BB showed reduced efficacy against strain Ei9. GA exhibited comparatively weaker disinfectant properties, and notably, PVP-I failed to demonstrate significant antimicrobial activity against any of the pathogens even at concentrations up to 1,000 mg/L.

**Table 3:** Minimal inhibitory concentrations of seven disinfectants against the 10 pathogenic bacteria.

Experimental disinfectants (mg/L)	Ah0548	Ah296	AvB1	Av0503	AsH1	AsoH2	EtZ1	Ei9	Pa22	Yr36
MBT	0.2	0.2	0.2	0.2	0.2	0.2	0.2	0.1	0.2	0.4
PVP-I	>1,000	>1,000	>1,000	>1,000	>1,000	>1,000	>1,000	>1,000	>1,000	>1,000
H_2_O_2_	32	32	24	24	24	24	72	24	24	24
PAA	1.5	1.5	1	1.5	1	1	5	1	1.5	1.5
BB	40	40	20	20	20	20	20	5	40	20
TCCA	40	40	40	40	40	40	40	20	40	60
GA	60	60	60	60	80	80	40	40	100	60

**Table 4:** Minimal bactericidal concentrations of seven disinfectants against the 10 pathogenic bacteria.

Experimental disinfectants (mg/L)	Ah0548	Ah296	AvB1	Av0503	AsH1	AsoH2	EtZ1	Ei9	Pa22	Yr36
MBT	0.2	0.2	0.4	0.2	0.2	0.2	0.8	0.4	0.2	0.4
PVP-I	>1,000	>1,000	>1,000	>1,000	>1,000	>1,000	>1,000	>1,000	>1,000	>1,000
H_2_O_2_	40	40	24	24	24	24	72	24	64	64
PAA	1.5	1.5	1.5	1.5	1	1	5	1	2	5
BB	40	40	40	20	20	20	20	10	80	20
TCCA	40	40	40	40	40	40	60	40	60	60
GA	120	120	140	120	120	100	140	120	140	140

### Bactericidal efficacy of MBT and TCCA on Ah0548

The bactericidal efficacy of MBT and TCCA against strain Ah0548 was evaluated through suspension tests, as summarized in Tables 5, 6. MBT exhibited concentration-dependent activity; 0.005 mg/L failed to achieve stable and significant bactericidal effects, whereas 0.01 mg/L demonstrated time-dependent bactericidal activity upon prolonged contact. Notably, this concentration achieved a > 3 log reduction in four out of five experimental replicates following 24 h exposure. Partial bactericidal effects were observed at a 6-h contact time, with two out of five trials showing >1 log reduction (Table 5).

**Table 5:** Efficacy of a range of concentrations of MBT against the test strain Ah0548.

Concentration (mg/L)	Contact time (h)	Reduction in viability (lgR) at test concentrations indicated				
		Test 1	Test 2	Test 3	Test 4	Test 5
0.005	1	NE	NE	NE	NE	NE
	6	0.95	NE	NE	NE	NE
	12	1	NE	NE	NE	NE
	24	1.91	NE	0.72	NE	NE
0.01	1	NE	NE	NE	NE	NE
	6	1.22	0.54	1.06	0.72	0.80
	12	2.65	1.75	2.04	2.55	2.41
	24	>4.18	3.54	4.34	3.99	>4.41
0.015	1	NE	NE	NE	NE	NE
	6	2.10	1.60	1.5	2.17	2.09
	12	3.57	3.10	3.00	3.42	3.33
	24	>4.18	4.45	4.34	>4.46	>4.41
0.02	1	NE	NE	NE	NE	NE
	6	3.90	2.82	2.43	3.07	3.13
	12	>4.18	>4.45	3.87	>4.46	4.41
	24	>4.18	>4.45	>4.34	>4.46	4.41
0.04	1	NE	NE	NE	NE	NE
	6	4.18	>4.45	>4.34	3.99	4.41
	12	>4.18	>4.45	>4.34	>4.46	4.41
	24	>4.18	>4.45	>4.34	>4.46	4.41

NE, no effect (lgR<0.5).

**Table 6:** Efficacy of a range of concentrations of TCCA against the test strain Ah0548.

Concentration (mg/l)	Contact time	Reduction in viability (lgR) at test concentration indicated				
		Test 1	Test 2	Test 3	Test 4	Test 5
0.05	30 min	NE	NE	NE	NE	NE
	1 h	NE	NE	NE	NE	NE
	6 h	NE	NE	NE	NE	NE
0.1	30 min	NE	NE	NE	NE	NE
	1 h	NE	NE	NE	NE	NE
	6 h	>4.48	NE	1.97	NE	NE
0.2	30 min	NE	NE	NE	0.87	0.77
	1 h	3.96	3.52	2.33	3.49	3.91
	6 h	>4.48	>4.42	4.30	>4.45	>4.39
0.4	30 min	>4.48	4.12	>4.3	>4.45	>4.39
	1 h	>4.48	>4.42	>4.3	>4.45	>4.39
	6 h	>4.48	>4.42	>4.3	>4.45	>4.39
0.6	30 min	>4.48	4.12	>4.3	>4.45	>4.39
	1 h	>4.48	>4.42	>4.3	>4.45	>4.39
	6 h	>4.48	>4.42	>4.3	>4.45	>4.39

NE, no effect (lgR<0.5).

In contrast, TCCA displayed more rapid bactericidal action at a concentration of 0.2 mg/L. This disinfectant achieved a > 4 log reduction within 6 h in four out of five trials, and notably, a > 3 log reduction was observed within just 1 h of contact in the majority (4/5) of experimental replicates (Table 6).

### Scale-trauma immersion infection model

**Figure 1:**
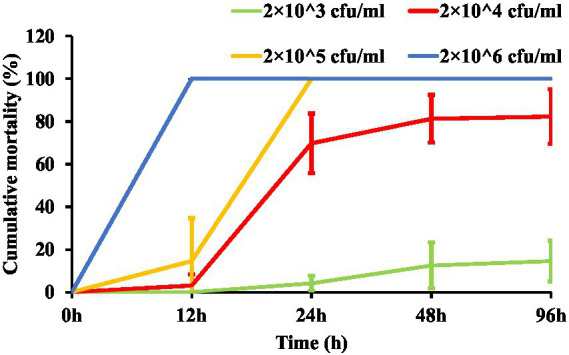
Cumulative mortality dynamics of zebrafish in the scale-trauma immersion infection model.

The cumulative mortality of zebrafish in the experimental infection model is shown in Figure 1. Only one individual died in the scale-trauma control group, while the uninfected control group maintained 100% survival throughout the 96-h observation period.

The 2 × 10^4^ cfu/mL challenge group exhibited time-dependent mortality rates of 69.79 ± 13.90%, 81.25 ± 11.2%, and 82.29 ± 12.80% at 24, 48, and 96 h post-infection, respectively, establishing this concentration as optimal for subsequent protection assays. Lower (2 × 10^3^ cfu/mL) and higher inocula (2 × 10^5^ and 2 × 10^6^ cfu/mL) were excluded due to suboptimal mortality profiles (insufficient lethality and excessive mortality, respectively).

**Figure 2:**
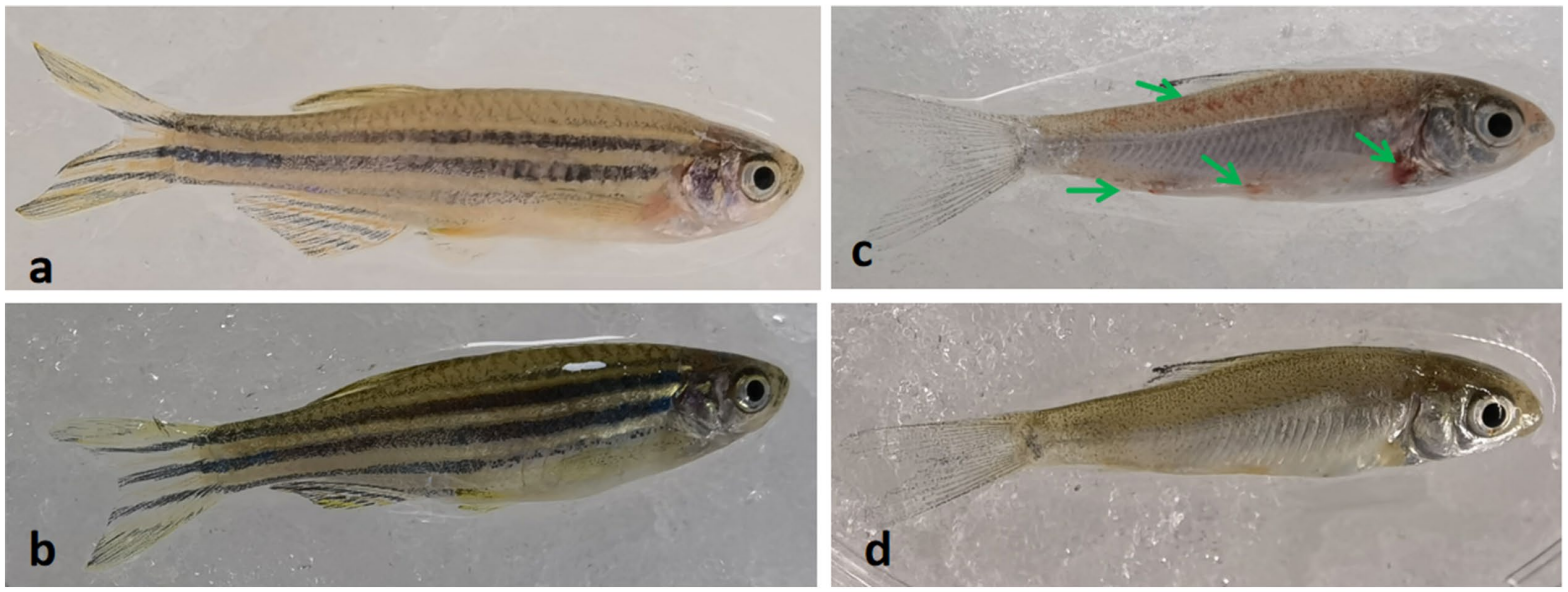
Morphological changes in zebrafish and crucian carp following Ah0548 infection via scale-trauma model. **(a)** Zebrafish infection group: scale-traumatized zebrafish challenged with Ah0548 (no disinfectant); **(b)** MBT-treated zebrafish: scale-traumatized zebrafish treated with 0.01 mg/L methylene bis(thiocyanate) (MBT) prior to bacterial challenge; **(c)** Crucian carp infection group: scale-traumatized crucian carp challenged with Ah0548 (no disinfectant); **(d)** MBT-treated crucian carp: scale-traumatized crucian carp treated with 0.01 mg/L MBT prior to bacterial challenge. Green arrows indicate hemorrhagic lesions on the dorsal musculature and at the fin base (crucian carp only).

**Figure 3:**
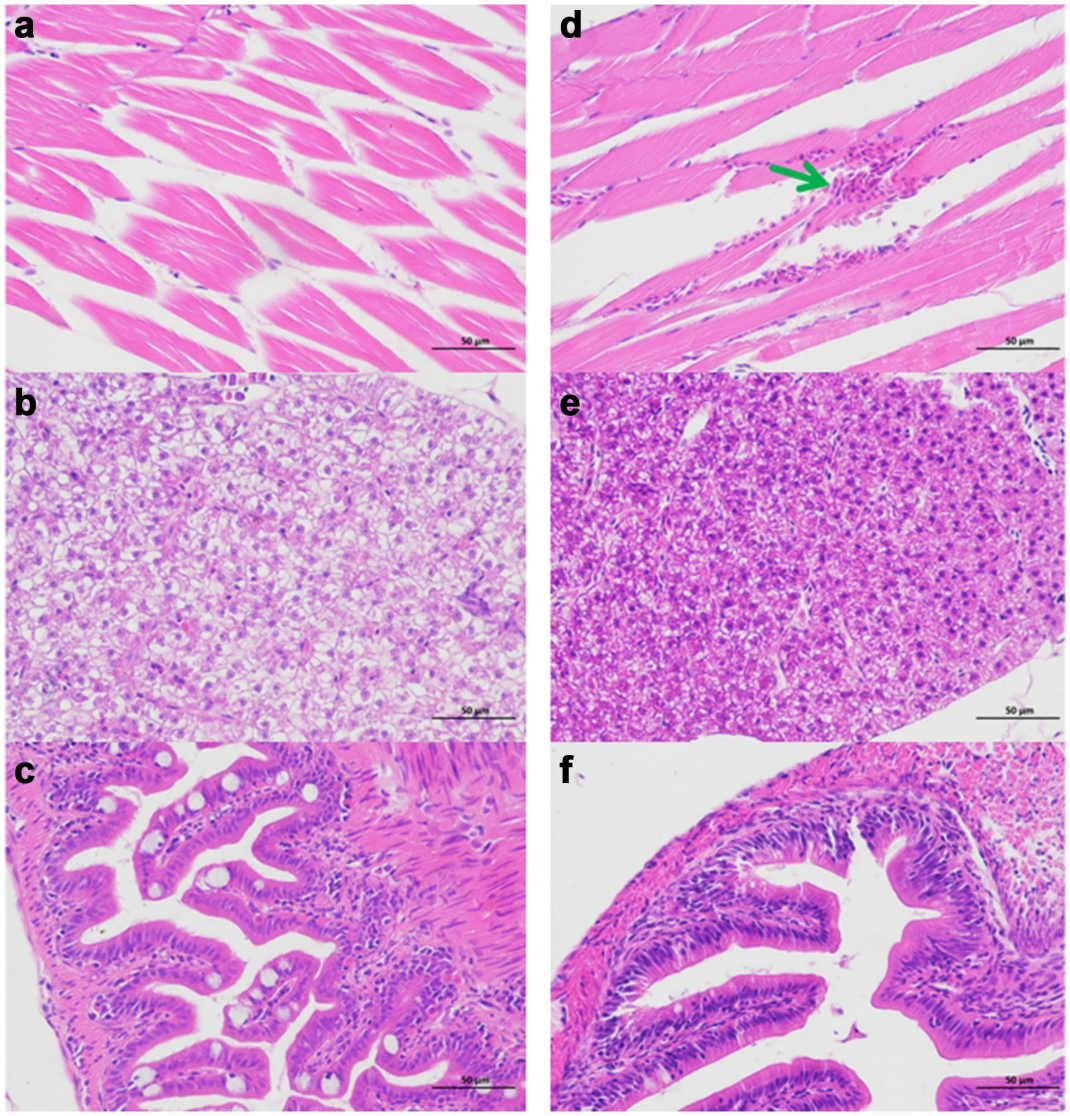
Histopathological analysis of Ah0548-infected zebrafish and crucian carp (×400). **(a)** Zebrafish muscle. **(b)** Zebrafish liver. **(c)** Zebrafish intestine. **(d)** Crucian carp muscle. **(e)** Crucian carp liver. **(f)** Crucian carp intestine. Green arrow denotes hemorrhagic focus in muscular tissue.

Comparative analysis revealed that crucian carp exhibited more pronounced disease manifestations than zebrafish under identical infection conditions. Infected crucian carp developed characteristic hemorrhagic presentations at scale-fin junctions (Figure 2c) and dorsal musculature (Figures 2c, 3d), whereas these pathologies were absent in preventively treated (Figure 2d). Notably, zebrafish displayed neither fin-base nor dorsal hemorrhages in either infected or treated groups (Figures 2a,b). Histopathological evaluation revealed preserved hepatic and intestinal architecture in both species, with no significant tissue abnormalities detected (Figure 3).

### Comparative protective efficacy of MBT and TCCA against *A. hydrophila* infection

**Figure 4:**
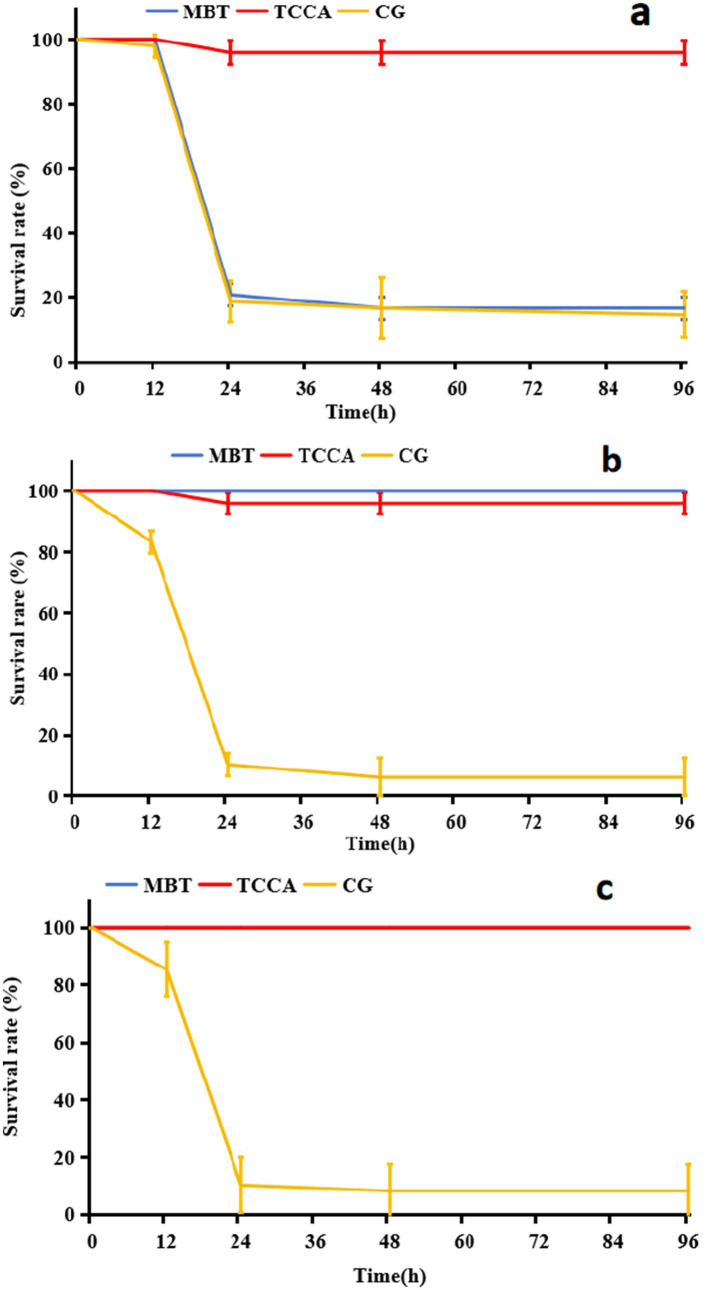
Comparative 96-h survival rates of zebrafish following exposed to Ah0548 (10^4^ cfu/mL) after treatment with MBT (0.01 mg/L) and TCCA (0.2 mg/L) for different disinfection durations. CG (infection control group): scale-traumatized zebrafish challenged with Ah0548 without disinfectant treatment; MBT: MBT treatment group; TCCA: TCCA treatment group. **(a)** 1 h, **(b)** 6 h, and **(c)** 12 h.

**Figure 5:**
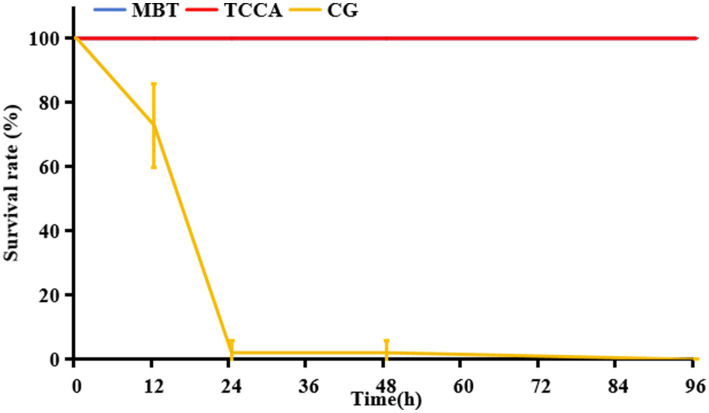
Comparative 96-h survival rates of crucian carp following exposure to Ah0548 (10^4^ cfu/mL) after 6-h disinfection with MBT (0.01 mg/L) and TCCA (0.2 mg/L). CG (infection control group): Scale-traumatized crucian carp challenged with Ah0548 (no disinfectant); MBT, MBT treatment group; TCCA, TCCA treatment group.

Both MBT and TCCA effectively prevented *A. hydrophila* infection in zebrafish when applied at concentrations of 0.01 mg/L and 0.2 mg/L, respectively; however, exposure duration significantly affected their protective efficacy (Figure 4). After 1 h of contact, the TCCA group exhibited strong protection, with a 96-h survival rate of 95.83 ± 3.61%. In contrast, the MBT group showed no significant protective effect, with a survival rate of 16.67 ± 3.61%, which was not significantly different from that of the infection group (14.58 ± 7.22%, *p* > 0.05) (Figure 4a). After 6 h of exposure, MBT achieved complete protection (100% survival), and TCCA maintained a survival rate of 95.83 ± 3.61% at 96 h. A similar trend was observed in the crucian carp model (Figure 5), where both disinfectants achieved 100% survival after 6 h of exposure. Furthermore, after 12 h of exposure, both MBT and TCCA provided full protective efficacy.

**Figure 6:**
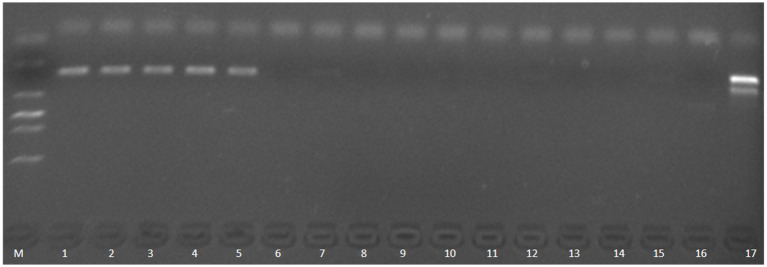
PCR verification of *aerA* gene in the infection group of zebrafish. M, DNA ladder (2000 bp marker); 1–5: infection control group (no disinfectant treatment); 6–8: TCCA treatment group (0.2 mg/L); 9–11: MBT treatment group (0.01 mg/L); 12–14: MBT control group (0.01 mg/L MBT, no bacterial exposure); 15–16: TCCA control group (0.2 mg/L TCCA, no bacterial exposure); 17: Posivtive control, genomic DNA from Ah0548 control.

Molecular validation using PCR targeting the aerolysin gene (*aerA*) confirmed successful *A. hydrophila* colonization in infected control groups, whereas both MBT and TCCA effectively prevented *A. hydrophila* infection (Figure 6).

### Acute toxicity comparison in zebrafish and Crucian carp exposed to MBT and TCCA

The acute toxicity parameters of MBT and TCCA are summarized in Table 7. Comparative analysis revealed that MBT exhibited higher acute toxicity than TCCA. Additionally, species-specific sensitivity was observed, with crucian carp displaying significantly greater susceptibility to MBT (LC₅₀: 0.0146 mg/L) compared to zebrafish (LC₅₀: 0.0364 mg/L).

**Table 7:** LC_50_ and SC of zebrafish and crucian carp exposed to MBT and for different periods.

Disinfectants	Animals	24 h		48 h		72 h		96 h		SC
		LC_50_ mg/L	95% confidence interval	LC_50_ mg/L	95% confidence interval	LC_50_ mg/L	95% confidence interval	LC_50_ mg/L	95% confidence interval	mg/L
MBT	Zebrafish	0.530	0.499–0.563	0.454	0.202–2.094	0.403	0.299–0.590	0.364	0.340–0.391	0.0364
	Crucian carp	0.297	0.245–0.365	0.184	0.158–0.0.217	0.162	0.152–0.173	0.146	0.137–0.156	0.0146
TCCA	Zebrafish	0.764	0.670–0.881	0.700	0.623–0.793	0.681	0.595–0.788	0.677	0.597–0.773	0.0677
	crucian carp	0.739	0.531–1.150	0.648	0.522–0.931	0.627	0.502–0.810	0.594	0.482–0.751	0.0594

## Discussion

A stable immersion-infection model serves as a critical foundation for *in vivo* evaluation of disinfectant bactericidal efficacy

Although intraperitoneal or intramuscular injections (Neely et al., 2002; Sun et al., 2024; Wang et al., 2024) are widely used infection models, they may not fully replicate natural disease processes. The skin serves as the primary entry point for fish pathogens, with intact skin acting as a protective barrier against bacterial invasion, while surface wounds or mucus layer removal facilitate infection (Chu and Lu, 2008; Neely et al., 2002; Dien et al., 2022). Pressley et al. (2005) demonstrated that adult zebrafish were not susceptible to static-immersion challenge unless epithelial layer was mechanically disrupted through scraping prior pathogen exposure. Similarly, Zou et al. (2023) established a reliable trauma-challenge model in zebrafish, though the pathogenicity observed in this model was lower than that induced via intraperitoneal injection.

In this study, exposure to *A. hydrophila* at 1.0 × 10^4^ cfu/mL induced >60% cumulative mortality in both zebrafish and crucian carp. While the immersion challenge model did not produce visible infection symptoms or significant histopathological alterations in zebrafish, crucian carp exhibited hemorrhagic lesions. These differences may be attributed to species-specific susceptibility, ontogenetic stage,or strain-specific pathogenicity (Dien et al., 2022; Semwal et al., 2023).

### Both MBT and TCCA effectively control *A. hydrophila* infection, with MBT demonstrating superior suitability for aquaculture water disinfection

PVP-I, H_2_O_2_, BB, TCCA, and GA are approved for use as disinfectants in China. PAA has also demonstrated effective bactericidal activity against certain pathogens (Meinelt et al., 2015). However, PVP-I showed no bactericidal or inhibitory activity even at concentrations up to 1,000 mg/L. GA exhibited the lowest inhibitory and bactericidal activity among the tested agents, except for PVP-I. While the antimicrobial efficacy of PAA and H_2_O_2_ against *A. hydrophila* was confirmed experimentally, their broader application in aquaculture remains limited due to thermodynamic instability and high costs (Farooq et al., 2025). BB showed moderate inhibitory and bactericidal effects, but chlorine-based disinfectants remain the mainstay choice for disease control in aquaculture.

TCCA, a conventional chlorine-based oxidant, has been widely used in aquaculture disinfection for decades. However, its efficacy and practical application are significantly influenced by organic matter (Jütte et al., 2023). Previous studies indicate that at a concentration of 0.405 mg/L (3 times the recommended dosage), TCCA achieved only 65.04% bactericidal efficacy in pond water matrices (Zhang et al., 2023). Consistent with these findings, our study revealed the MBC of TCCA reached 40 mg/L—two orders of magnitude higher than its effective bactericidal concentration (0.2 mg/L) in the quantitative suspension test. Furthermore, the increasing resistance to traditional disinfectants presents challenges for biosecurity control (Tong et al., 2021), underscoring the urgent need for new disinfectants or alternative solutions to replace those currently in widespread use.

In the protective tests, 0.01 mg/L MBT effectively prevented and controlled *A. hydrophila* infection. In contrast, the MBC of MBT against two *A. hydrophila* strains was 0.2 mg/L—20 times the concentration required for bactericidal efficacy in the suspension test—indicating that MBT may be less affected by organic matter than TCCA. Notably, Chen et al. (2021) study reported an MBC of 2.93 mg/L, 14.65 times higher than the observed value in our study (0.2 mg/L), likely due to differences in MBT preparation methods. Since *A. hydrophila* infection is typically associated with water containing a high organic load (Abdella et al., 2024), MBT is more suitable than TCCA for preventing and controlling *A. hydrophila* infection due to its less affected by organic matter.

Additionally, at its minimal bactericidal concentration, MBT required an exposure time of 6 h to eliminate *A. hydrophila*, compared to only 1 h for TCCA. This difference is likely due to variations in their bactericidal mechanisms. This relatively slow onset time highlights the need to develop improved dosage forms to enhance the practicality of MBT in aquaculture. Furthermore, once bacterial infections are detected, the timely application of MBT may help limit pathogen transmission through water and enhance overall disease control efficacy.

### The lower effective concentration of MBT ensures its feasibility for controlling *A. hydrophila*

As a novel disinfectant, both its effectiveness and toxicity should be considered. Short-term toxicity evaluations in rainbow trout (*Oncorhynchus mykiss*) demonstrated 14-day and 60-day LC_50_ values of 0.084 mg/L and 0.065 mg/L, respectively, for MBT (Maas-Diepeveen and van Leeuwen, 1988). Furthermore, embryonic-larval teratogenicity assays indicated a no-observed-effect concentration of 0.032 mg/L in the same species (Maas-Diepeveen and van Leeuwen, 1988). In our study, the SCs of MBT for zebrafish and crucian carp were determined to be 0.0364 and 0.0146 mg/L, respectively. Although the effective concentration (0.01 mg/L) was lower than the SC for both species, it was approached the SC threshold for crucian carp. Therefore, careful attention should be paid to dosing when applying MBT to cyprinid species to minimize the risk of toxicity to cultured animals.

In addition MBT degradation in aquatic environments produces thiocyanate ions and formaldehyde (Chen et al., 2020). The environmental fate, persistence, and potential aquatic toxicity of these degradation products—particularly thiocyanate ions—require further investigation. As a newly proposed disinfectant for aquaculture applications, MBT also warrants comprehensive assess its long-term toxicity and environmental risks.

Notably, MBT exhibited the lowest MIC and MBC values among all seven tested disinfectants against the evaluated bacterial strains (Tables 3, 4). This indicates its potential not only for controlling *A. hydrophila,* but also for combating a broad spectrum of economically important aquatic pathogens (Tables 3, 4). To the best of our knowledge, this study provides the first documented evidence of MBT’s potent bactericidal activity against this broad spectrum of aquatic bacterial pathogens, highlighting its value disinfectant in aquaculture.

## Conclusion

Both TCCA and MBT effectively control *A. hydrophila* infection; however, MBT demonstrates superior suitability due to its effectiveness at a lower dosage (0.01 mg/L). Although MBT exhibits higher toxicity to target aquatic species, its effective concentration remains lower than SC values, suggesting minimal risk at recommended dosages. Moreover, MBT shows promise for preventing and controlling not only *A. hydrophila* but also other aquatic bacterial pathogens (Tables 3, 4), underscoring its potential as a valuable biosecurity agent in commercial aquaculture practices.

## Data Availability

The raw data supporting the conclusions of this article will be made available by the authors, without undue reservation.
